# Camelid reporter gene imaging: a generic method for *in vivo* cell tracking

**DOI:** 10.1186/s13550-014-0032-8

**Published:** 2014-06-26

**Authors:** Lode RY Goethals, Tomas J Bos, Luc Baeyens, Frank De Geeter, Nick Devoogdt, Tony Lahoutte

**Affiliations:** 1 In Vivo Cellular and Molecular Imaging Laboratory, Vrije Universiteit Brussel, Laarbeeklaan 103, Jette 1090, Belgium; 2 Department of Radiology, Universitair Ziekenhuis Brussel, Laarbeeklaan 101, Jette 1090, Belgium; 3 Department of Cellular and Molecular Medicine, UC San Diego, 9500 Gilman Drive, La Jolla 92093, CA, USA; 4 Beta Cell Neogenesis, Vrije Universiteit Brussel, Laarbeeklaan103, Jette 1090, Belgium; 5 Department of Nuclear Medicine, Universitair Ziekenhuis Brussel, Laarbeeklaan 101, Jette 1090, Belgium

**Keywords:** Cell tracking, Fluorescence, Bioluminescence, SPECT/CT, Nanobody, YFP

## Abstract

**Background:**

To combine the sensitivity of bioluminescent imaging (BLI) with the 3D and quantitative properties of pinhole single-photon emission computed tomography (SPECT)/micro-computed tomography (CT) (phSPECT/micro-CT), we generated stable cell lines that express a yellow-fluorescent protein (YFP) and Gaussia luciferase (GLuc) fusion protein (YFP/GLuc). For *in vivo* phSPECT detection of this YFP/GLuc protein, a nanobody, targeted against yellow and green fluorescent proteins (anti-YFP-Nb), was site specifically labelled with ^99m^Tc.

**Methods:**

Human embryonic kidney cells (HEK293T) were cultured and passaged every 3 days. 10E5 cells were transduced with YFP/GLuc-containing vector: both membrane-targeted (MT-YFP/GLuc) and non-targeted (YFP/GLuc) fusion proteins were developed. These vectors were compared against a SKOV-3 cell line stably expressing green fluorescent-firefly luciferase (GFP/Fluc) and HEK293T cells expressing red fluorescent protein in combination with a Gaussia luciferase (Red/GLuc). Transduction efficiencies were scored by fluorescence microscopy, and transduced cells were enriched by fluorescence-activated cell sorting (FACS). GLuc and FLuc functionality was tested *in vitro* by list-mode BLI. Subsequently, cells were transplanted subcutaneously in athymic (nu/nu) mice (MT-YFP/GLuc: *n* = 4, YFP/GLuc: *n* = 6, GFP/FLuc: *n* = 6, Red/GLuc: *n* = 4). Labelling efficiency of anti-YFP-Nb was measured using instant thin layer chromatography. One week after transplantation, ^99m^Tc-labelled anti-YFP-Nb was injected intravenously and pinhole (ph) SPECT/micro-CT was performed, followed by *in vivo* BLI.

**Results:**

Cells showed high levels of fluorescence after transduction. The cells containing the MT-YFP/GLuc were positive on fluorescence microscopy, with the fluorescent signal confined to the cell membrane. After cell sorting, transduced cells were assayed by BLI and showed a significantly higher light output both *in vitro* and *in vivo* compared with non-transduced HEK293T cells. The anti-YFP-Nb labelling efficiency was 98%, and subsequent phSPECT/micro-CT demonstrated visible cell binding and significantly higher transplant-to-muscle ratio for both the MT-YFP/GLuc and YFP/GLuc transplanted cells, compared with the GFP/FLuc and Red/GLuc group.

**Conclusion:**

This study provides a proof of principle for a nanobody-based cell tracking method, using a YFP/GLuc fusion protein and anti-YFP-Nb in a model of subcutaneously transplanted transduced HEK293T cells.

## Background

A growing number of animal and preclinical studies reveal the potential of cell-based therapies in a variety of applications [[1]], including myocardial infarction and beta-cell precursor transplantation in diabetes. To evaluate the long-term efficacy of these experimental treatments, it is useful to monitor transplanted cells qualitatively and quantitatively *in vivo*. Indirect cell labelling through reporter gene imaging provides an elegant method to perform such cell tracking [[2]]. Reporter genes are available for different imaging modalities, which can be largely divided into optical imaging, MRI and nuclear imaging (PET/SPECT). Optical cell tracking exploits the sensitivity of liquid-cooled CCD cameras with the inherently low background signal to produce a high sensitivity (up to 10E − 17 mol/l) [[3]]. In contrast, luminescent signals are difficult to quantify and suffer a high attenuation of visible light, especially when emitted from the inner organs. In parallel, nuclear imaging modalities generate a signal that combines high sensitivity (up to 10E − 12 mol/l), with quantitative 3D possibilities, when using adequate scatter and attenuation corrections [[3]–[5]]. Commonly used SPECT- or positron emission tomography (PET)-based reporter gene systems include herpes simplex virus-thymidine kinase (HSV-tk)-, sodium iodine symporter (NIS)- and mutant dopamine receptor (D_2_R)-based systems [[6]–[8]].

This study investigates the feasibility of developing a new multimodality cell tracking method, combining the optical sensitivity and the quantitative possibilities of nuclear imaging with the advantageous physico-chemical properties of nanobodies. Nanobodies are the antigen-binding fragments from heavy-chain-only antibodies naturally occurring in Camelidae [[9]]. Nanobodies are considered to be the smallest intact antigen-binding fragments derived from a functional antibody. Their limited size of approximately 15 kDa and their general robustness make them very suitable for *in vivo* imaging [[10]]. Nanobodies labelled with ^99m^Tc have been used for targeting specific epitopes *in vivo*, for example, CEA and EGFR2 [[11],[12]].

In this study, we used an anti-green fluorescent protein (GFP) nanobody, labelled with ^99m^Tc, to perform *in vivo* cell tracking. This nanobody is cross-reactive with both yellow fluorescent protein (YFP) and GFP, two closely related fluorescent proteins [[13],[14]]. As molecular target, we transduced cells lentivirally with a fusion protein, consisting of a yellow fluorescent protein/Gaussia luciferase (YFP/GLuc) fusion protein. This fusion protein combines the fluorescence of YFP, a bright and versatile fluorescent protein (excitation peak 514 nm, emission peak 527 nm), with Gaussia luciferase, a naturally secreted luciferase, cloned from Gaussia princeps with coelenterazine as its substrate. The oxidation of coelenterazine does not require ATP, and it is therefore also suitable for imaging when the protein is secreted or bound to the outer cell membrane [[15]].

To facilitate recognition of the fluorescence epitope by the nanobody, we generated a vector that displays the YFP/GLuc fusion protein on its outer membrane [[16]] and compared it against its intracellular counterpart. Both were compared against a combined GFP/firefly luciferase (FLuc)-expressing cell line. Firefly luciferase has been extensively utilized for *in vivo* bioluminescence and catalyses the oxidation of d-luciferin to yield light with a peak emission of 562 nm in the presence of O_2_, magnesium and ATP. Throughout this study, a red fluorescent protein/Gaussia luciferase-expressing HEK293T cell line (Red/GLuc) was used as a negative control.

In summary, the aim of this study was to provide a proof of principle for the monitoring of transplanted cells *in vivo* using intravenously injected ^99m^Tc-labelled anti-YFP nanobodies.

## Methods

### Plasmids

**Figure 1:**
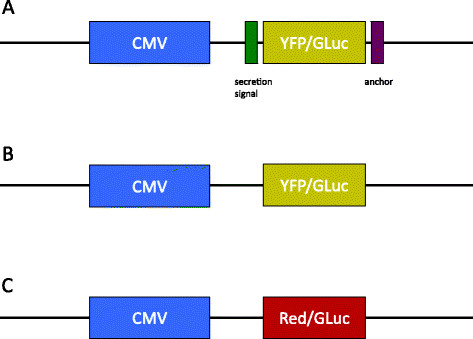
Viral constructs encoding for the fusion proteins MT-YFP/GLuc (A), YFP/GLuc (B) and Red/GLuc (C).

Lentiviral constructs were generated using standard molecular biology techniques (Figure 1). The YFP/GLuc fusion protein was cloned into a lentiviral backbone (LV.YFP/GLuc). The pDisplay™ vector (Life Technologies, Invitrogen, Carlsbad, CA, USA), a vector that anchors any protein to the cell membrane, was cloned into this YFP/GLuc lentiviral backbone, yielding the LV.MT-YFP/GLuc vector. A control vector was prepared by cloning the GLuc gene into a lentiviral plasmid containing a red fluorescent fluorochrome (Red/GLuc).

### Cell lines

Human embryonic kidney (HEK) 293T cells were cultured in Dulbecco's modified Eagle medium (DMEM), 10% fetal bovine serum (FBS) and penicillin-streptomycin and passaged every 3 days. Cells were grown at 37°C and in a 5% CO_2_ atmosphere. A total of 100,000 cells were transduced with all three of the aforementioned lentiviral vectors, and 2 days upon transduction, cells were sorted for fluorescence expression and subcloned cells were cultured according to the above-mentioned protocol. A GFP and firefly luciferase (FLuc)-expressing SKOV3 cell line was purchased (Bio-Connect®, Huissen, the Netherlands).

### Cell line validation *in vitro*: microscopy and *in vitro* BLI

Transduction efficiency was scored by fluorescence microscopy. Further localization of the fluorescent signal was obtained by confocal microscopy (Zeiss LSM710 NLO TiSa multiphoton confocal microscope using Zeiss Zen2011 software, Carl Zeiss NV-SA, Zaventem, Belgium).

Luciferase functionality was determined using *in vitro* bioluminescence imaging (BLI). HEK293T and SKOV3 cells were plated in six-well plates. For GLuc-expressing cells, a coelenterazine solution (50 μl per well of a 1 mg/ml solution) was added to approximately 2E6 cells per well. In parallel, 2E6 FLuc-expressing SKOV3 cells were imaged upon adding 150 μl of d-luciferin (30 mg/ml) per well. Images were obtained with a Photo Imager camera (Biospace, Paris, France) that allows list-mode acquisition.

### Xenograft model

All animal experiments were performed with the approval of the ethical committee for animal research of the Vrije Universiteit Brussel. During transplantation, all mice were anaesthetized with a mixture of oxygen and 5% isoflurane and maintained with a mixture of oxygen and 2.5% isoflurane. Twenty immune-deficient athymic (nu/nu) mice were purchased (Charles River, Chatillon-sur-Chalaronne, France). Cells growing exponentially in culture were suspended in 150 μl of phosphate-buffered saline (PBS) and 150 μl Matrigel (BD Biosciences, San Jose, CA, USA) and transplanted subcutaneously in the hind leg. Mice were inoculated with either MT-YFP/GLuc-expressing cells (*n* = 4), YFP/GLuc-expressing cells (*n* = 6) or cells that expressed Red/GLuc (*n* = 4). Also, six mice were transplanted with the SKOV3 cells. All transplants consisted of 2E6 cells.

### *In vivo* BLI

BLI was performed immediately after SPECT/CT while the animals were still anaesthetized. Coelenterazine (1 mg/ml) or d-luciferin (30 mg/ml) was injected intravenously via the tail vein. A solution of 30 μl substrate and 120 μl NaCl was administered. Immediately after substrate injection, mice were imaged using a Photo Imager camera (Biospace, France). Light emission was measured using the large field-of-view setting and registered using the photon counting technology during 1 min. A grey-scale photographic image of the mice was fused with the bioluminescent images. The most intense luciferase signal is shown in red, the weakest signal in blue. To analyse the images, an elliptical region of interest (ROI) was drawn over the transplant location, using a constant surface area.

### Nanobody labelling

Nanobodies were labelled with ^99m^Tc at their hexahistidine tail. For the labelling, [^99m^Tc(H_2_O)_3_(CO)_3_]^+^ was synthesized by adding 1 ml of ^99m^TcO_4_^−^ (0.84 to 3.7 GBq) to an Isolink kit (Mallinckrodt Medical BV, Petten, the Netherlands) containing 4.5 mg of sodium boranocarbonate, 2.85 mg of sodium tetraborate, 10 mg H_2_O, 8.5 mg of sodium tartrate and 7.15 mg of sodium carbonate at pH 10.5. The vial was incubated at 100°C in a boiling bath for 20 min. The freshly prepared [^99m^Tc(H_2_O)_3_(CO)_3_]^+^ was allowed to cool at room temperature for 5 min and neutralized with 125 μl of 1 M HCl to pH 7 to 8. A total of 500 μl of the tricarbonyl solution was added to 50 μl of carbonate buffer at pH 8. The mixture was incubated for 90 min at 60°C in a water bath. The ^99m^Tc-nanobody solution was purified on a NAP-5 column (GE Healthcare, Little Chalfont, Buckinghamshire, UK) pre-equilibrated with PBS. The labelling efficiency was determined by instant thin layer chromatography.

### Pinhole SPECT/micro-CT

One week after transplantation, anaesthesia was induced with isoflurane gas 3% in an air/oxygen mixture. For the induction of anaesthesia, ketamine/xylazine was given intraperitoneally. The animal was placed in supine position during acquisition, and images were acquired 60 min after tracer injection. Micro-CT imaging was followed by pinhole SPECT on separate systems.

Micro-CT was performed using a dual-source CT scanner (Skyscan 1178 micro-CT, Skyscan, Kontich, Belgium) with a 60-kV voltage and 615-mA tube current at a resolution of 83 μm. The total body scan time was 2 min. Images were reconstructed using filtered back projection (NRecon; Skyscan).

The SPECT acquisitions were performed using a dual-headed gamma camera (e.cam, Siemens Medical Solutions, Hoffman Estates, IL, USA) equipped with a triple pinhole collimator. The collimator has a focal length of 250 mm. Sixty-four projections of each 30 s were acquired over 360° of rotation into a 64 × 64 matrix. The total imaging time was approximately 15 min.

To guide image fusion of the SPECT with the micro-CT, two acrylic circular disks containing 3 3.7 MBq ^57^Co point sources incorporated in organic ion exchange beads of 1-mm diameter (Canberra, Zellik, Belgium) were firmly fixed to the scanner bed. The disks measured 25 mm in diameter and 3 mm in thickness. The six beads provided reference points in both imaging modalities and were used as markers to generate a spatial transformation matrix. The spatial position of the six fiducial markers was determined manually using A Medical Image Data Examiner (AMIDE).

phSPECT images were analysed using AMIDE [[17]]. 3D elliptical regions of interest were drawn around the transplants on SPECT/micro-CT images. Transplant to muscle ratios were calculated as the mean activity within these ROIs divided by the mean activity within ROIs drawn in muscle tissue.

### *In vitro* biodistribution

After imaging, all animals were killed. Blood, liver, kidneys, muscle and transplants were dissected and weighed. The amount of activity was determined using a gamma counter (Canberra, Zellik, Belgium). Tracer uptake was expressed as the percentage injected dose per gram of tissue (%ID/g).

### Statistical analysis

Average transplant-to-muscle ratios were compared between groups using the Kruskall-Wallis test. Post-hoc comparisons were made by Mann-Whitney tests using a Bonferroni correction for multiple testing.

## Results

### Transduction efficiency

Fluorescence-activated cell sorting (FACS) showed a positive fraction of 78% in the MT-YFP/GLuc group. Within the YFP/GLuc group, 63% was positive. In the Red/GLuc group, 62% was positive. These positive fractions were sorted and subcultured.

### Cell line validation *in vitro*: microscopy and *in vitro* BLI

**Figure 2:**
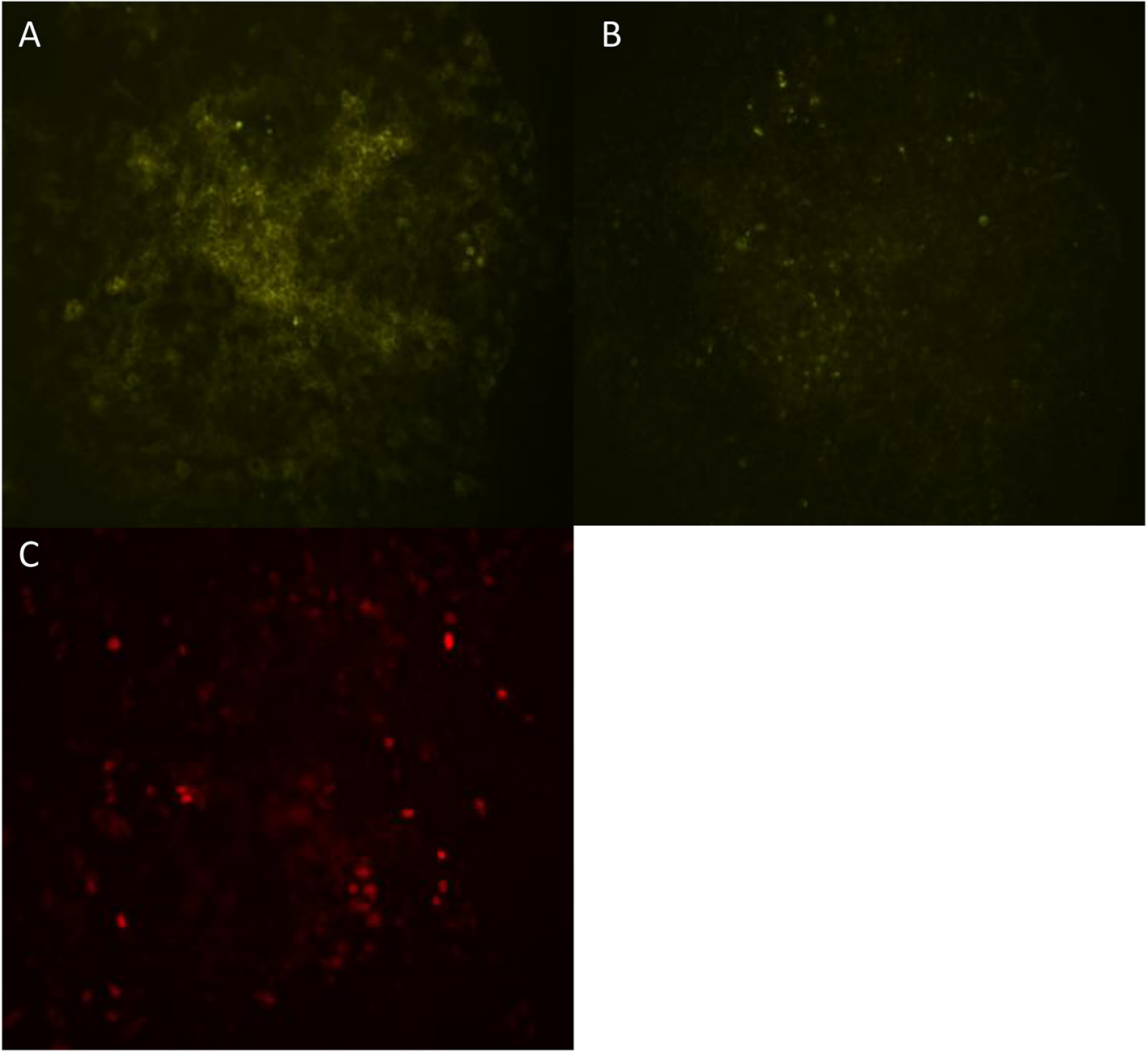
Fluorescence microscopy of MT-YFP/GLuc (A), YFP/GLuc (B) and Red/GLuc (C) cell lines.

**Figure 3:**
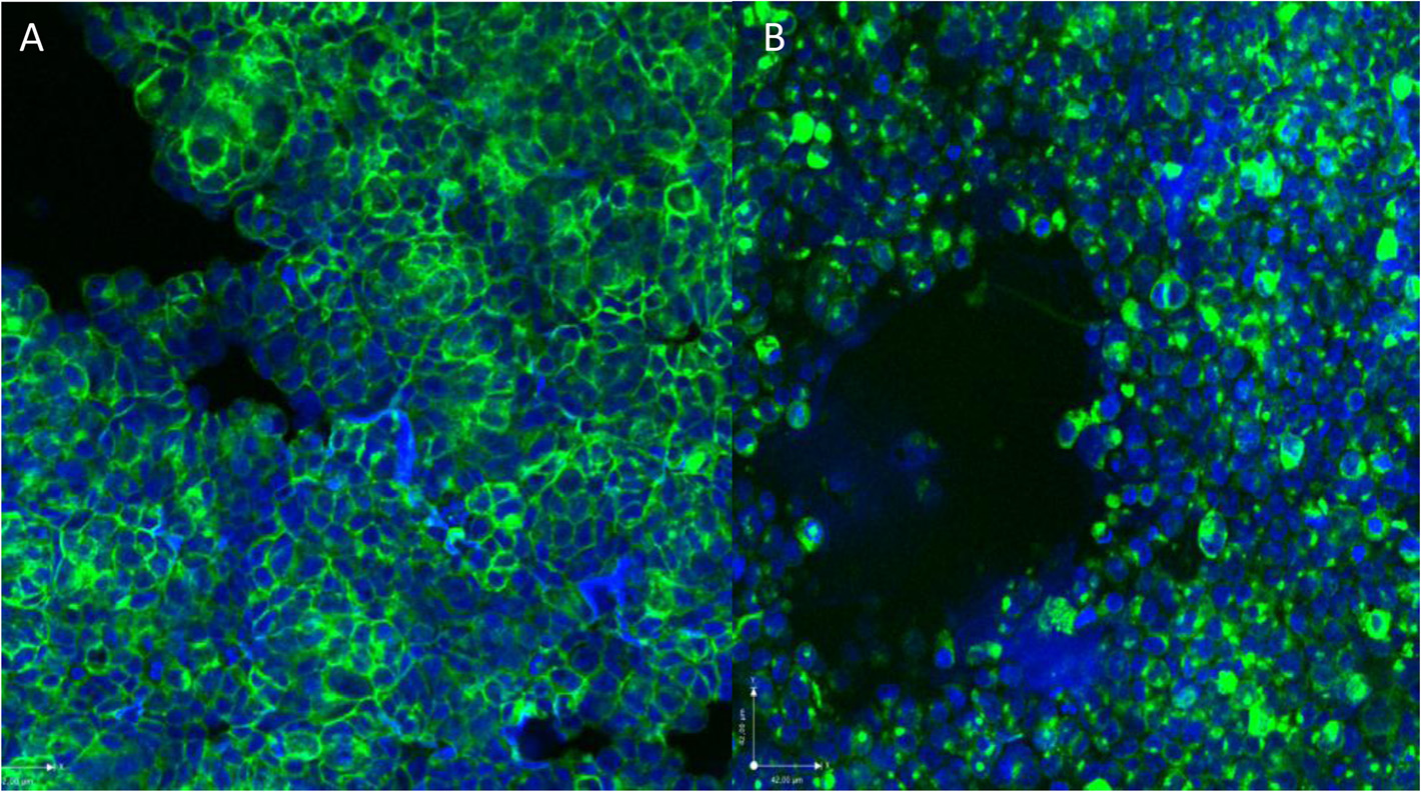
Confocal microscopy of MT-YFP/GLuc (A) and YFP/GLuc (B) cell lines, further localizing the fluorescent signal.

Functionality of the fluorescence in the in-house developed cell lines was shown using fluorescence microscopy (Figure 2B,C). Confocal microscopy showed fluorescence confined to the cell membrane in the MT-YFP/GLuc cell line, suggesting proper targeting of protein to the cell membrane (Figure 3A). In the YFP/GLuc cell line, the signal emanated from the cytoplasm as well as the cell membrane and possibly even from the extracellular space.

**Figure 4:**
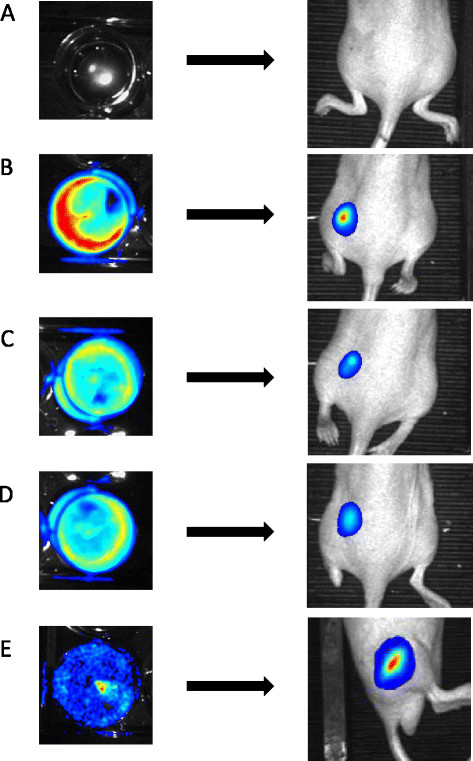
***In vitro*** **and** ***in vivo*** **BLI.** BLI was performed in the non-transduced HEK293T cell line **(A)**, the MT-YFP/GLuc **(B)**, YFP/GLuc **(C)**, Red/GLuc **(D)** and GFP/FLuc **(E)**. All images are scaled to the same maximum.

*In vitro* BLI showed an increased light output in all cell lines, compared with non-transduced HEK293T cells (Figure 4A,B,C,D,E).

### *In vivo* BLI

*In vivo* BLI showed increased light output in all transduced transplants compared with the non-transduced transplant. This suggests viable transplants in every transduced cell line (Figure 4A,B,C,D,E).

### Nanobody labelling efficiency

Labelling efficiency of the anti-YFP nanobodies was in excess of 98%.

### Pinhole SPECT/micro-CT

**Figure 5:**
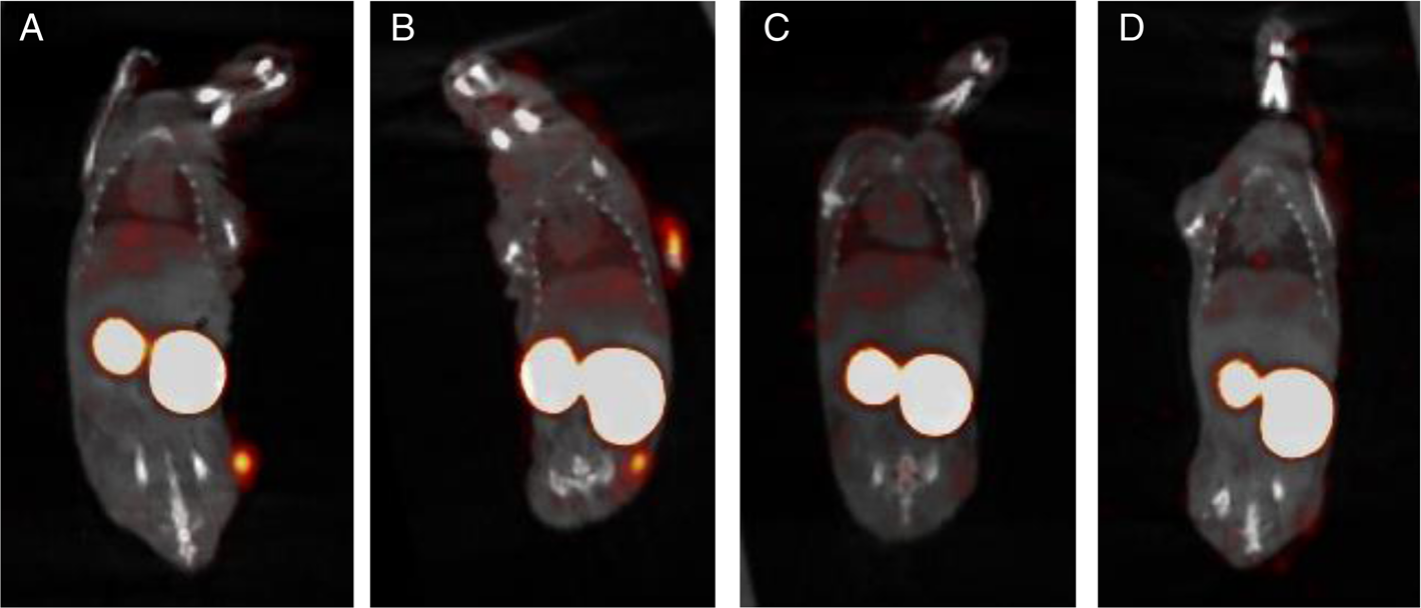
Para-coronal SPECT/micro-CT fused images at the level of the transplants in the MT-YFP/GLuc (A), YFP/GLuc (B), Red/GLuc (C) and GFP/Fluc (D).

A well-delineated spot of tracer accumulation was found on the sites of inoculation in transplants in the MT-YFP/GLuc and the YFP/GLuc groups, representing binding of the nanobody on the transplants. In the GFP/FLuc and the Red/GLuc groups, no tracer uptake could be discerned within the transplants (Figure 5A,B,C,D). Image processing yielded an average transplant-to-muscle ratio of 0.85 ± 0.2 in the Red/GLuc group, 7.59 ± 1.34 in the MT-YFP/GLuc group, 5.65 ± 0.8 in the YFP/GLuc group and 1.1 ± 0.4 in the GFP/FLuc group. Kruskall-Wallis testing showed a significance between groups. Post-hoc testing using multiple Mann-Whitney tests further localizes these differences between the YFP/GLuc and the GFP/GLuc groups (*p* < 0.001) and between the GFP/GLuc and the Red/GLuc (*p* < 0.001). Significance was also achieved between the MT-YFP/GLuc group compared with the Red/GLuc group (*p* < 0.001) and the GFP/GLuc group (*p* < 0.001). No significance was noted between YFP/GLuc and MT-YFP/GLuc, nor between the Red/GLuc and GFP/FLuc.

### *In vitro* biodistribution

Dissection results for the transplants, expressed as %ID/g, are given in Table 1, confirming transplant targeting in the MT-YFP/GLuc and the YFP/GLuc groups, but not in the Red/GLuc or the GFP/FLuc group.

**Table 1:** Dissection data

	**Transplant (%ID/g)**	**Muscle (%ID/g)**	**Liver (%ID/g)**	**Blood (%ID/g)**	**Kidneys (%ID/g)**
MT-YFP/GLuc	2.2 ± 0.9	0.2 ± 0.1	0.4 ± 0.2	0.2 ± 0.1	20.3 ± 7.3
YFP/GLuc	1.8 ± 0.9	0.1 ± 0.1	0.4 ± 0.2	0.3 ± 0.1	20.4 ± 6.1
Red/GLuc	0.2 ± 0,1	0.1 ± 0.1	0.5 ± 0.2	03 ± 0.1	22.8 ± 6.2
GFP/FLuc	0.3 ± 0.2	0.2 ± 0.1	0.4 ± 0.3	0.6 ± 0.2	30.2 ± 9.8

For MT-YFP/GLuc (*n* = 4), YFP/GLuc (*n* = 6), Red/GLuc (*n* = 4) and GFP/FLuc (*n* = 6) groups.

## Discussion

In this study, we developed a fusion protein, comprising the yellow fluorescent protein (YFP), linked to Gaussia luciferase (GLuc), as a molecular target for ^99m^Tc-labelled anti-YFP nanobodies.

To allow epitope recognition, we targeted this fusion protein to the cell membrane using the pDisplay™ vector, a 5.3-kb mammalian expression vector that allows display of proteins on the cell surface, using a fusion of the protein at the N-terminus of the Ig κ chain leader sequence, which directs the protein to the secretory pathway, and the C-terminus of the platelet-derived growth factor receptor transmembrane domain, which anchors the protein to the plasma membrane, displaying it on the extracellular side [[16]].

The total size of this multimodal reporter system is approximately 50 kDa, leaving the expression cassette small enough to be carried in most viral vectors [[16]].

Viral vectors encoding for two or even three proteins, generated through recombinant DNA technology, have been described, combining for example GFP with thymidine kinase (TK) or FLuc with red fluorescent protein and TK, separated by an intra-ribosomal entry site (IRES) sequence. These combinations yield relatively long expression cassettes (up to 3.7 kb in the case of FLuc-RFP-TK sequence) and suffer loss of activity for the reporter downstream of the IRES element [[18]]. To avoid this loss of activity, we used the fusion protein YFP-GLuc rather than an IRES sequence separated vector. Fusion of proteins can lead to inactivation of one or both parts of the fusion protein [[18]]. Throughout this study, the fusion of GLuc and YFP preserved the bioluminescent and fluorescent properties.

Localization of the fluorescent signal on confocal microscopy showed a fluorescent signal confined to the cell membrane in the MT-YFP/GLuc group. In the non-targeted YFP/GLuc group, the fluorescent signal was more heterogeneously distributed, as it originated from the cytoplasm as well as from the cell membrane and possibly even from the extracellular space. The YFP/GLuc epitope was recognized *in vivo* by its ^99m^Tc-anti-YFP nanobody in both the MT-YFP/GLuc group and the YFP/GLuc group, with a higher transplant-to-muscle ratio in the MT-YFP/GLuc group. A possible explanation for these findings is found in the GLuc part of the YFP/GLuc protein. Since GLuc DNA includes a putative signal peptide sequence for secretion, GLuc naturally enters the secretory pathway [[19]]. In the absence of membrane anchorage, this might lead to portions of the fusion protein on the membrane as well as intracellularly and extracellularly. This hypothesis is strengthened by the lack of binding of the anti-YFP nanobody onto the GFP/FLuc cell line since FLuc acts as an intracellular luciferase and reacts with its substrate d-luciferin intracellularly [[20]].

This study is the first to exploit the advantageous imaging properties of nanobodies for *in vivo* nuclear imaging in a context of reporter gene imaging, in combination with luminescence and fluorescence. Because of these nanobody characteristics, especially the small size (approximately 15 kDa), transplants are clearly distinguished on phSPECT/CT with average transplant-to-muscle ratios of 7.59 as soon as 45 min after injection of the tracer. Although comparison with other tracers is difficult, Venisnik et al. achieved a similar %ID within their transplants using an engineered anti-carcinoembryonic antigen (CEA) antibody fused to a Gaussia luciferase. In our study, we show a faster kinetics compared with Venisnik et al. [[21]]. Undoubtedly, this faster *in vivo* kinetics owes to the small size of the nanobody in comparison with a Gaussia luciferase-engineered anti-CEA antibody [[21]].

During the past years, several reporter systems have been developed that generate SPECT or PET contrast. The most well known are the HSV-tk-, D_2_R- or NIS-based systems. Each of these methods has its inherent advantages and disadvantages. HSV-tk-based imaging systems suffer high liver and intestinal background activity due to hepatobiliary excretion of tracers such as ^18^F-FHPG [[22]]. The D_2_R-based ^99m^Tc-TRODAT SPECT system has a low dynamic range and yields only modest signal to background activities due to the lipophilicity of the tracer [[23]]. NIS-expressing transplants use ^99m^Tc or ^123^I as tracer, with physiological uptake in the stomach, thyroid and glandular tissue. Efflux of isotopes from the cells due to lack of organification and low uptake of tracers can compromise adequate cell tracking [[24]]. Intravenously injected radiolabelled nanobodies have a specific biodistribution characterised by a high renal excretion, in combination with a low background signal in other organs such as the liver, lungs and myocardium. This allows high signal-to-noise ratios using this nanobody-based imaging method for the long-term assessment of cell therapy.

In the present study, the anti-YFP nanobody was labelled with ^99m^Tc, but labelling of nanobodies with PET radio-isotopes (^18^F or ^68^Ga) is feasible [[25]]. In addition, labelling of antibody fragments with fluorescent labels as well as MRI contrast is feasible [[16]], rendering this YFP-anti-YFP reporter system applicable for multimodal *in vivo* imaging studies.

## Conclusion

We provided proof of principle for a YFP-GLuc fusion protein as reporter protein to perform noninvasive cell tracking, in combination with a ^99m^Tc-labelled anti-YFP nanobody. Both the membrane-targeted and the non-targeted lentiviral vector encoding for a YFP/GLuc fusion protein could be targeted *in vivo*.

## Competing interests

The authors declare that they have no competing interests.

## Authors' contributions

LG (the first author) conceived and coordinated the study and carried out the experiments. TB constructed the lentiviral vectors for transduction. LB performed fluorescence and confocal microscopy. FD performed statistical analysis and participated in the design of the study. ND prepared the nanobodies. TL participated in the design and coordination of the study and helped to draft the manuscript. All authors read and approved the final manuscript.
